# Waist circumference and waist-to-height ratio are associated with periodontal pocketing—results of the Health 2000 Survey

**DOI:** 10.1186/s12903-017-0336-y

**Published:** 2017-01-18

**Authors:** Sanna Kangas, Petra Timonen, Matti Knuuttila, Antti Jula, Pekka Ylöstalo, Anna-Maija Hannele Syrjälä

**Affiliations:** 1 Private Dental Office Viisaudenhammas, Rovaniemi, Finland; 2 Dental Training Clinic, Social and Health Services, Oulu, Finland; 3 Medical Research Center Oulu, Oulu University Hospital and University of Oulu, Oulu, Finland; 4 National Institute for Health and Welfare (THL), Helsinki, Finland; 5 Periodontology and Geriatric Dentistry, Unit of Oral Health Sciences Research, P.O. Box 5281, FI-90014 Oulu, Finland; 6 Institute of Dentistry, University of Eastern Finland, Kuopio Campus, Kuopio, Finland; 7 Oral and Maxillofacial Department, Kuopio University Hospital, Kuopio, Finland

**Keywords:** Periodontal disease, Periodontal pocketing, Waist-to-height ratio, Waist circumference, Overweight

## Abstract

**Background:**

Body mass index (BMI) has been found to associate with different parameters of chronic periodontal disease in previous studies. It is reasonable to expect that central adiposity measures, such as waist circumference and waist-to-height ratio, which indirectly takes into account visceral fat, are more accurate measures of obesity-related oral health risks than BMI. The aim of this study was to examine whether central obesity is associated with periodontal pocketing, an indication of infectious chronic periodontal disease.

**Methods:**

The study was based on a subpopulation from the national Health 2000 Survey in Finland. It included dentate, non-diabetic, never-smoking subjects aged 30–49 (*n* = 1287). The outcome variable was the number of teeth with deepened periodontal pockets (4 mm or more) and the number of teeth with deep periodontal pockets (6 mm or more). Central obesity was measured by means of waist circumference (WC) and waist-to-height ratio (WHtR). Poisson regression models were used to estimate prevalence rate ratios (PRR) and their 95% confidence intervals.

**Results:**

Our main finding was that both WC and WHtR were associated with the number of teeth with deeper (4 mm or more) periodontal pockets; the PRR for the fifth quintile in WC was 1.5, CI: 1.2–1.9 and in WHtR 1.4, CI: 1.1–1.7, when compared to the lowest quintile. Corresponding figures for deep (6 mm or more) periodontal pockets were 2.3, CI: 0.9–6.1 for WC and 1.9, CI: 0.8–4.4 for WHtR. There were no essential differences in the strengths of the associations between WC and WHtR and the number of teeth with deepened periodontal pockets.

**Conclusion:**

Both central adipose measures—WC and WHtR—seem to be associated with periodontal pocketing in non-diabetic, never-smoking subjects aged 30–49 years old.

## Background

A large number of non-experimental studies have reported a weak or moderately strong association between overweight or obesity and a variety of parameters of periodontal disease [1–5]. In these studies, overweight and obesity have most often been measured by means of the body mass index (BMI), but also other measures of adiposity such as waist circumference (WC), waist-hip ratio (WHR) and waist-to-height ratio (WHtR) have been used.

A well-known shortcoming of BMI is that it does not take into account body composition nor distribution of fat. Regarding the distribution of fat tissue, it has been observed that visceral fat accumulation has harmful health effects; it increases the risk of cardiovascular disease more than does subcutaneous fat [6] and it increases the risk of cardiovascular disease regardless of BMI [7]. In some periodontal studies, adiposity measures such as WC and WHR that take visceral fat into account have been reported to be associated more strongly with different parameters of infectious periodontal diseases than commonly used BMI [8–11]. These findings suggest that adiposity measures that measure visceral fat accumulation accurately indicate the obesity-related health risks for periodontal health, at least more accurately than commonly used BMI.

The simplest measure of central adiposity is waist circumference, and it has been shown to be a fairly reliable indicator for periodontal disease [8, 12, 13]. However, an obvious shortcoming of waist circumference (WC) is that it relies solely on the person’s waist circumference. Based on this it is expected that WHtR, which also takes into account the person’s height, would be a better measure than waist circumference alone. WHtR has recently been reported to reveal several health risks including diabetes, hypertension, dyslipidaemia, metabolic syndrome and cardiovascular diseases better than BMI and WC, although in some cases the differences were small and statistically insignificant [14]. To date, only one study has analysed WHtR in relation to periodontal disease; a longitudinal study found that WHtR predicts periodontal disease progression better than BMI [15].

We have reported earlier that BMI is associated with periodontal pocketing in the Health 2000 Survey [16, 17]. Based on the known shortcomings of BMI, our aim in the present paper was to study whether central adiposity measures—WC and WHtR—are consistently associated with the number of teeth with deepened periodontal pockets, an indication of an infectious form of chronic periodontal disease among never-smoking, non-diabetic, 30–49-year-old persons.

## Methods

### Study design

The National Institute for Health and Welfare (formerly the National Public Health Institute of Finland) performed a Health 2000 Survey between 2000 and 2001. Data for the Health 2000 Survey were obtained from laboratory measurements, clinical health and oral examinations, self-administered questionnaires and interviews. The original survey was a nationally representative sample and consisted of 8028 subjects aged 30 or older living in continental Finland.

This cross-sectional study was restricted to subjects aged 30–49 years old. The exclusion of diabetic persons yielded a population of 2856 subjects. After further exclusion of persons who had smoked, there were 1326 persons left; of those 1297 subjects had periodontal data and 1287 subjects had both periodontal data and central obesity and height measurements available.

The clinical oral surveys were carried out by five field units (each including one dentist and one dental nurse or hygienist). They examined the condition of the periodontium and teeth using a headlamp, a mouth mirror and a WHO periodontal probe in line with WHO instructions. More information about the Health 2000 Examination Survey is reported by Heistaro [18].

Participation in this survey was voluntary and the participants gave their written consent for this study. The Ethical Committee for Epidemiology and Public Health of the Hospital District of Helsinki and Uusimaa approved the study protocol.

### Outcome variables

The outcome variables were the number of teeth with deepened periodontal pockets (4 mm or more) and the number of teeth with deep periodontal pockets (6 mm or more). Periodontal pocket depth was probed on four surfaces (distobuccal, mid-buccal, mid-oral and mesio-oral) of each tooth, except third molars, and the deepest measurement on each tooth was recorded. There was 82% agreement (κ = 0.32) between a reference examiner and the field examiners [19]. For this outcome (the number of teeth with deepened periodontal pockets) no power-calculation was made.

### Explanatory variables

Information on weight and height was obtained from the clinical health examination. Waist circumference (WC, in centimetres) and waist-to-height ratio (WHtR; waist circumference in centimetres divided by height in centimetres) were used as explanatory variables. Men and women were separately categorised into five categories according to the distribution of WC and WHtR, and then respective categories for men and women were combined in non-gender-specific analyses (Table 3).

### Other variables

In this study, gender, age and educational level were used to describe sociodemographic background. Educational level was classified into three categories: the highest level consisted of those who had a university degree or had graduated from a polytechnic, the second level consisted of those who had graduated from high school and the lowest-level subjects had less than a high school education.

The presence of dental plaque was recorded on one side on three indicator teeth (the most posterior tooth on the upper right side, and the most posterior tooth and canine on the lower left side) [18]. The results were categorised into three categories using a modified version of the method described by Sillness and Löe [20]: no dental plaque on the indicator teeth, dental plaque in the gingival margins on the indicator teeth, and dental plaque also elsewhere on the indicator teeth. Dental visits were classified into two categories: regular *vs.* irregular check-ups. Toothbrushing was categorised into three categories: at least twice a day, daily and more seldom.

BMI (Body Mass Index; body weight in kilograms divided by the square of height in metres) was used as a categorised variable according to the WHO definition [21] of overweight and obesity: BMI < 25, 25–29.9 and ≥ 30. Lipid-lowering medication was classified into three groups: yes, no or unknown. Alcohol consumption was measured as grams of alcohol per week.

The basic characteristics of the study population as well as the basic characteristics of the study population by quintiles of waist circumference are presented in Table 1.

**Table 1:** Characteristics of the study population according to quintiles of waist circumference

	Waist circumference					
	Total *n* = 1287	I quintile *n* = 266 (lowest)	II quintile *n* = 255	III quintile *n* = 236	IV quintile *n* = 266	V quintile *n* = 264 (highest)
Sociodemographic variables						
Gender, proportion of males, %	42.5	42.4	42.6	41.0	43.9	42.2
Age, mean (± SD)	39.6 (±5.7)	37.5 (±5.2)	39.3 (±5.6)	39.0 (±5.9)	41.2 (±5.3)	40.9 (±5.7)
Education						
Low, %	11.9	7.8	12.4	10.0	13.3	15.7
Intermediate, %	36.9	29.7	32.2	42.3	38.7	41.9
High, %	51.2	62.5	55.4	47.7	48.0	42.3
Dental variables						
Number of teeth						
Mean (± SD)	27.1 (±3.9)	28.0 (±3.1)	27.1 (±4.2)	27.5 (±3.1)	26.9 (±3.9)	26.3 (±4.8)
Median (interquartile range)	28 (3)	28 (3)	28 (2)	28 (2)	28 (2)	28 (4)
Min, max	1, 32	4, 32	1, 32	9, 32	6, 32	3, 32
Number of teeth with periodontal pockets ≥ 4 mm						
Mean (± SD)	2.6 (±4.0)	1.8 (±2.9)	2.5 (±3.7)	2.2 (±3.5)	2.7 (±4.0)	3.6 (±5.2)
Median (interquartile range)	1 (3)	1 (3)	1 (4)	1 (3)	1 (4)	1 (5)
Min, max	0, 28	0, 14	0, 25	0, 20	0, 26	0, 28
Number of teeth with periodontal pockets ≥ 6 mm						
Mean (± SD)	0.2 (±1.2)	0.1 (±0.5)	0.1 (±0.5)	0.1 (±0.7)	0.2 (±0.9)	0.5 (±2.4)
Median (interquartile range)	0.00 (0)	0.00 (0)	0.00 (0)	0.00 (0)	0.00 (0)	0.00 (0)
Min, max	0, 26	0, 4	0, 7	0, 7	0, 9	0, 26
Dental plaque						
No dental plaque, %	40.7	46.1	43.1	46.3	38.9	29.8
Dental plaque at the gingival margins, %	50.2	46.7	48.4	45.9	52.9	56.5
Dental plaque also elsewhere, %	9.1	7.2	8.4	7.8	8.1	13.6
Dental visits						
Regular check-ups, %	69.0	75.1	71.0	70.9	67.7	60.3
Irregular, %	31.0	24.9	29.0	29.1	32.3	39.7
Toothbrushing						
At least twice a day, %	68.7	78.2	71.9	64.8	71.6	56.2
Daily, %	27.1	18.3	26.9	31.7	24.1	35.3
Less often, %	4.2	3.4	1.2	3.5	4.2	8.5
Alcohol consumption, mean (± SD)	60.3 (±97.7)	44.2 (±58.7)	66.1 (±97.7)	56.8 (±94.9)	67.0 (±101.6)	67.5 (±123.1)
BMI, mean (± SD)	25.7 (±4.4)	21.5 (±1.8)	23.4 (±1.9)	24.9 (±1.7)	26.6 (±2.0)	31.9 (±4.2)
Lipid-lowering medication, %	1.2	0.0	0.8	1.3	1.5	2.6

### Statistical methods

Medians, interquartile ranges and minimum and maximum values for the number of teeth, the number of teeth with deepened periodontal pockets (4 mm or more) and the number of teeth with deep periodontal pockets (6 mm or more) were calculated.

Prevalence rate ratios (PRR) and 95% confidence intervals (95% CI) were estimated using Poisson regression models. Covariates in the regression models included potential determinants of periodontitis such as age, gender, educational level, presence of dental plaque, toothbrushing frequency, dental visits and use of lipid-lowering medication. Stratified analyses according to gender were performed.

A stratified two-stage cluster sampling design was applied in the study. The sample was weighted by post-stratification according to gender, age and region. The data analyses were performed using STATA 8.0 to take into account the two-stage cluster sampling design.

## Results

The unadjusted and adjusted prevalence rate ratios (PRR) with 95% confidence intervals (CI) are shown in Tables 2 and 3.

**Table 2:** Relation of study variables to the number of teeth with periodontal pockets

	Teeth with periodontal pockets ≥ 4 mm	Teeth with periodontal pockets ≥ 6 mm
	Unadjusted PRR (95% CI)	Unadjusted PRR (95% CI)
Waist circumference (*n* = 1287)		
I quintile	1	1
II quintile	1.4 (1.1–1.8)	1.1 (0.4–2.9)
III quintile	1.2 (0.9–1.6)	1.3 (0.4–3.7)
IV quintile	1.5 (1.1–2.0)	2.3 (0.8–6.2)
V quintile	2.1 (1.7–2.7)	6.0 (2.6–13.9)
Waist-to-height Ratio (*n* = 1287)		
I quintile	1	1
II quintile	1.3 (0.9–1.7)	1.1 (0.4–2.5)
III quintile	1.3 (1.0–1.7)	1.2 (0.5–3.0)
IV quintile	1.6 (1.2–2.1)	3.2 (1.3–7.5)
V quintile	2.0 (1.5–2.5)	5.9 (2.7–12.6)
Sociodemographic variables		
Gender (*n* = 1297)		
Female	1	1
Male	0.7 (0.6–0.8)	0.4 (0.2–0.7)
Age (*n* = 1297)		
30–34	1	1
35–39	1.3 (1.0–1.7)	2.4 (0.9–6.5)
40–44	1.6 (1.2–2.1)	5.6 (2.0–16.1)
45–49	2.0 (1.5–2.7)	5.2 (1.9–13.9)
Education (*n* = 1297)		
High	1	1
Intermediate	1.3 (1.1–1.5)	3.3 (1.7–6.3)
Low	1.9 (1.4–2.4)	7.2 (3.6–14.3)
Dental variables (*n* = 1297)		
Number of teeth		
≥ 25	1	1
21–24	1.5 (1.2–2.0)	2.4 (1.1–4.9)
1–20	1.9 (1.2–2.8)	3.6 (1.5–8.9)
Dental plaque (*n* = 1294)		
No dental plaque	1	1
Dental plaque at the gingival margins	2.1 (1.7–2.6)	2.7 (1.2–6.3)
Dental plaque also elsewhere	4.9 (3.7–6.6)	19.4 (7.8–48.8)
Dental visits (*n* = 1253)		
Regular check-ups	1	1
Irregular	1.5 (1.2–1.8)	3.7 (2.0–6.9)
Toothbrushing (*n* = 1253)		
At least twice a day	1	1
Daily	1.1 (0.9–1.4)	2.0 (0.9–4.5)
Less often	2.1 (1.5–2.9)	3.8 (1.8–8.0)
Lipid-lowering medication (*n* = 1297)		
Yes	1	1
No	0.8 (0.5–1.3)	2.9 (0.5–17.7)
Unknown	0.9 (0.5–1.5)	4.6 (0.5–43.9)

**Table 3:** Relation of WC and WHtR to the number of teeth with periodontal pockets

	WC	WHtR
	Adjusted PRR (95% CI)	Adjusted PRR (95% CI)
	*n* = 1241	*n* = 1241
Teeth with periodontal pockets ≥ 4 mm		
I quintile	1.0	1
II quintile	1.3 (1.0–1.7)	1.2 (0.9–1.5)
III quintile	1.2 (0.9–1.5)	1.2 (0.9–1.5)
IV quintile	1.2 (0.9–1.6)	1.2 (0.9–1.6)
V quintile	1.5 (1.2–1.9)	1.4 (1.1–1.7)
Teeth with periodontal pockets ≥ 6 mm		
I quintile	1.0	1
II quintile	0.8 (0.3–2.3)	0.6 (0.2–1.7)
III quintile	1.1 (0.4–3.1)	0.8 (0.3–1.9)
IV quintile	1.3 (0.5–3.7)	1.6 (0.7–4.0)
V quintile	2.3 (0.9–6.1)	1.9 (0.8–4.4)

Models are adjusted for age, gender, education, dental plaque, toothbrushing, dental visiting and lipid-lowering medication

*WC* Waist Circumference, *WHtR* Waist-to-height Ratio

As seen in Table 3, persons with a large WC and a high WHtR had, on average, a 40–60% higher likelihood of having teeth with periodontal pockets ≥ 4 mm than those belonging to the lowest quintile (reference category).

As seen in Table 3, persons with a large WC and a high WHtR were, on average, 30–50% more likely to have more teeth affected with pockets of more than 4 mm than those belonging to the lowest quintile (reference category). There were some deviations from linearity in the association between both WC and WHtR and the number of teeth with deepened periodontal pockets ≥ 4 mm.

The association with the number of teeth with periodontal pockets ≥ 6 mm was less consistent, showing pronounced deviations from linearity. Confidence intervals were wide and none of the risk estimates were statistically significant at a p-level of 0.05.

When comparing the associations between WC and WHtR and periodontal pockets ≥ 4 mm, there were no essential differences in the strengths of the associations (Table 3). The associations with deep periodontal pockets were difficult to compare due to the large confidence intervals, but the overall association between WHtR and deep periodontal pockets was somewhat weaker than that of WC (Table 3).

When men and women were analysed separately, the associations between both WC and WHtR and teeth with periodontal pockets of ≥ 4 mm in both men and women were close to those found in the total study population. There were inconsistent differences between genders in the association of WC and WHtR with the number of teeth with periodontal pockets ≥ 6 mm. Confidence intervals were large and the association showed pronounced deviations from linearity (data not shown).

## Discussion

We expected that the measure which also takes into account the person’s height would be associated more strongly with periodontal pocketing than would the simple measure of central obesity. However, to our surprise, the strength of the association between WHtR and periodontal pockets did not differ essentially from that of WC. We also expected that the WC and WHtR measures would be associated more strongly than BMI with periodontal disease. Again, to our surprise, the strength of the association was of quite the same magnitude as the association between BMI and periodontal pocketing, reported earlier by Ylöstalo and co-workers [16].

Based on the findings of the present study and the findings of the earlier one using the same data, we have to conclude that our results are somewhat different from a number of earlier studies, because the use of WHtR as a measure of obesity did not seem to provide any essential benefits compared with WC. The use of WC or WHtR did not provide any essential benefits compared with commonly used BMI, either. The reason why WC or WHtR were not superior to BMI could be that BMI may sufficiently indicate obesity-related health risks in this low-risk population. This may related to the fact that BMI appears to have a fairly high correlation with body fat among the young population, especially among women [22]. High correlations between BMI and other measures of adiposity have also been reported in these Health 2000 data [16].

There are currently a very limited number of studies where a multitude of adiposity measures including both WC and WHtR have been used. One of them is a study by Gorman and co-workers [15], who reported that WHtR was associated more strongly than WC with periodontal disease progression, measured by means of probing pocket depth, alveolar bone loss and clinical attachment loss. The fact that the results were not in line with previous studies may be related to the other properties of this study population; due to restrictions the study subjects were non-smokers and their mean age was less than 40 years. Because the data were restricted to persons who were less than 50 years old, we cannot, of course, say anything about how the central measures of obesity behave in older populations. However, habitual changes in body composition—increasing fat and decreasing muscle mass—suggest that measures which take into account these aspects are most likely better than simple measures such as absolute or relative weights, or even a simple measure of central obesity. This reasoning is supported by the study by Romero-Corral et al. [22], who reported that correlations between BMI and body fat are also somewhat lower in older age groups.

### Strengths and limitations

The effect of competing risks for periodontitis such as tobacco smoking, diabetes and age was eliminated by restricting this study to those who had never smoked, had no signs of diabetes and were 30–49 years old. The confounding effect of other potential risks for periodontitis was controlled by using multivariate models. Covariates that were used to control for the effects of poor oral hygiene were dental plaque and toothbrushing frequency. These methods controlled completely for the effects of diabetes and smoking, and also to some degree for the effects of various behavioural factors associated with smoking. The methods used to control for the effect of poor oral hygiene are always more or less incomplete, and it is possible that some residual confounding related to oral hygiene exists.

The outcome variables were continuous variables—the number of teeth with pocket depth ≥ 4 mm and ≥ 6 mm—both indicative of infectious periodontal diseases, although of different severity. Regarding its registration and measurement, it should be noted that the fact that registering was done at tooth level may have caused an underestimation of the extent of periodontal disease. Also the fact that pocket measurement was done on four predetermined sites may have caused an underestimation of periodontal disease. However, the effects of the above-mentioned aspects are mostly likely small, as most of the participants had a fairly healthy periodontium, i.e., they had a very small number of teeth with deepened periodontal pockets.

## Conclusions

Based on these data, it can be estimated that the subjects who belong to the highest quintiles of WC or WHtR have a 40–60% increased likelihood of having teeth with periodontal pockets at least 4 mm deep compared with those belonging to the lowest quintiles of WC or WHtR. The typical number of teeth with deepened periodontal pockets at least 4 mm deep was two or three among lean persons, but if the person had a large waist circumference, measured either absolutely (WC) or relatively (WHtR), he/she had approximately one tooth more with a deepened periodontal pocket. This excess of periodontally affected teeth, which can be attributed to central obesity, can be compared, for example, with the excess that can be attributed to daily smoking, which in these data was about 2–3 teeth with periodontal pocketing [16].

Both WC and WHtR seemed to be associated with periodontal pocketing, an indication for infectious chronic periodontal disease among non-diabetic, never-smoking subjects aged 30–49 years old. The overall interpretation is that the findings of this study lend support to the findings of previous studies where an association between obesity and various parameters of chronic periodontitis has been found. Self-evidently this study also lends support to the current view in periodontal research that obesity may have adverse effects on the periodontium. In the present study, we could not observe any essential differences in the strengths of the associations between WC and WHtR and periodontal pocketing, indicating that additional information about other dimensions of the body does not provide any essential benefits in this age group when assessing obesity-related risks for periodontal health.

## Abbreviations

BMI: Body mass index
CI: Confidence intervals
PRR: Prevalence rate ratios
WC: Waist circumference
WHR: Waist-hip ratio
WHtR: Waist-to-height ratio
